# A Circular, Wireless Surface-Electromyography Array

**DOI:** 10.3390/s24041119

**Published:** 2024-02-08

**Authors:** Kenneth Deprez, Eliah De Baecke, Mauranne Tijskens, Ruben Schoeters, Maarten Velghe, Arno Thielens

**Affiliations:** 1 Department of Information Technology, imec, Ghent University, 9052 Ghent, Belgium; kenneth.deprez@ugent.be (K.D.); eliah.debaecke@ugent.be (E.D.B.); mauranne.tijskens@ugent.be (M.T.); ruben.schoeters@ugent.be (R.S.); maarten.velghe@rivm.nl (M.V.); 2 Centre for Sustainability, Environment and Health, National Institute for Public Health and the Environment (RIVM), 3720 BA Bilthoven, The Netherlands; 3 Photonics Initiative, The Advanced Science Research Center, The Graduate Center of the City University of New York, New York, NY 10031, USA

**Keywords:** Surface-Electromyography, Bluetooth Low Energy, gesture recognition, Prosthetics

## Abstract

Commercial, high-tech upper limb prostheses offer a lot of functionality and are equipped with high-grade control mechanisms. However, they are relatively expensive and are not accessible to the majority of amputees. Therefore, more affordable, accessible, open-source, and 3D-printable alternatives are being developed. A commonly proposed approach to control these prostheses is to use bio-potentials generated by skeletal muscles, which can be measured using surface electromyography (sEMG). However, this control mechanism either lacks accuracy when a single sEMG sensor is used or involves the use of wires to connect to an array of multiple nodes, which hinders patients’ movements. In order to mitigate these issues, we have developed a circular, wireless s-EMG array that is able to collect sEMG potentials on an array of electrodes that can be spread (not) uniformly around the circumference of a patient’s arm. The modular sEMG system is combined with a Bluetooth Low Energy System on Chip, motion sensors, and a battery. We have benchmarked this system with a commercial, wired, state-of-the-art alternative and found an r = 0.98 (*p* < 0.01) Spearman correlation between the root-mean-squared (RMS) amplitude of sEMG measurements measured by both devices for the same set of 20 reference gestures, demonstrating that the system is accurate in measuring sEMG. Additionally, we have demonstrated that the RMS amplitudes of sEMG measurements between the different nodes within the array are uncorrelated, indicating that they contain independent information that can be used for higher accuracy in gesture recognition. We show this by training a random forest classifier that can distinguish between 6 gestures with an accuracy of 97%. This work is important for a large and growing group of amputees whose quality of life could be improved using this technology.

## 1. Introduction

Millions of patients are living with an amputation [1,2]. This includes approximately 4 million upper limb amputees. This is a growing number of patients who are faced with limitations in their daily activities due to their amputation [1,2]. One method of rehabilitation for these patients is the use of prostheses. A variety of upper limb prostheses exist that correspond to the type of amputation and desired functionality. This work focuses on transradial prostheses, i.e., artificial arms that attach below the elbow, suitable for patients with an amputation of the lower arm. However, the system is designed to be fully modular so that it can also be placed on the upper arm if needed. This type of prosthesis can be passive or active. A passive prosthesis only restores anthropomorphic appearance or can be changed manually from one gesture to another [3]. An active prosthesis has the ability to make movements with the aim of restoring hand functionalities. Advances in sensor and actuation technology, wireless communication, signal processing techniques, and computing hardware make such prostheses possible. However, usage of these devices remains limited [4] due to the relatively high cost [5], control difficulties, insufficient capabilities, and dexterity levels. Misclassifications of intended actions play a role in these control difficulties, which eventually lead to prostheses abandonment [6].

In order to lower the cost of these prostheses, there have been developments in the creation of open-source and low-cost models for transradial prostheses that can be manufactured by household 3D printers. However, at the moment, the control mechanisms for these types of prostheses lack the accuracy of their commercial counterparts. Surface electromyography (sEMG) is a commonly proposed sensory technique to control these devices. In this approach, electrodes worn on the surface of the skin record bio-potentials generated by skeletal muscles on the upper arm or the remaining part of the lower arm. This technique is relatively inexpensive, non-invasive, and (open-source) algorithms exist for gesture recognition using this technique [7]. Therefore, it is popular in the field. However, this control mechanism lacks accuracy when an sEMG sensor on a single on-body location is used [8] because the sensor is, in that case, mainly sensitive to potentials that originate from sources (muscles) closest to the sensor. Therefore, several recent works try to work with multiple sEMG nodes or an array of sEMG electrodes that are distributed along and/or around the arm [7]. This comes with the disadvantage that multiple wired connections between these nodes and the prosthesis impede patients’ comfort. 

The current state of the art in sEMG sensors does not provide an answer to the problems listed above because existing low-cost sEMG sensors are either wired, single node devices [9,10], wireless single nodes [11], or they are wired arrays [12]. Wireless sEMG sensors (arrays) are currently available commercially [9,13] but at a relatively high cost. Very recently, some experimental wireless, low-cost sEMG arrays have been demonstrated [14,15]. However, these arrays have taken the approach of working with a distributed array rather than working with an integrated collocated array, as is proposed here. 

To mitigate the issues listed above, the goal of this work is to develop a linear, collocated array in which all sEMG electrodes are placed along one single interconnect that is wrapped around the circumference of the arm, creating a circular array. Our second goal is to avoid a wired connection between the prosthetic arm and the sensors to offer flexibility in sensor placement and increase patient comfort. Finally, we want to do all of this at a low cost.

This work is part of a larger project in which we are developing a low-cost wireless prosthesis that can be controlled by a low-cost wireless sEMG array. This paper focuses on the array development. In order to use sEMG for the control of prostheses, a classification of the measured signals into the intended movements is necessary. Multiple classifiers are already available. Table 1 shows an overview of prior research on gesture classifiers for wearable sEMG sensors with multiple EMG channels. For each reference, we have listed the number and type of gestures that were studied, the number of sensors, the classification method used, the type of features that were used, and the obtained accuracy. It can be seen that for sampling rates above 0.5 kHz, sufficient accuracy for gesture recognition can be obtained [16], that time domain (TD) and frequency domain (FD) features can both be used for accurate classification [17] and that it is in general possible to develop a classifier using a multi-channel array. We are looking for a classifier that could be run on a mobile node placed either on the sEMG array or in a low-cost prosthesis. In order to show that this would be possible with the array that we developed in our manuscript, our third goal is to demonstrate that this array could be used for gesture recognition. While the overview shown in Table 1 is not exhaustive, it provides important information on what classifier we could develop to test its performance on our array.

In order to reach our goals, we developed a low-cost wireless module suitable for sEMG measurements and benchmarked it against a commercial low-cost sensor. This module is extended with three slave nodes that it can control and read through a bus system. Finally, it is shown that for a set of standardized gestures, the sEMG measurements of the array’s elements are uncorrelated, indicating that they contain independent information for gesture classification.

## 2. Materials and Methods

This section describes the methodology used to develop the different sEMG sensor prototypes, the gestures used for benchmarking and comparison with a commercial sensor, and the methods used for this benchmarking and comparison. Our sensor was benchmarked in comparison to a commercial, wired solution, and the functioning of our sensor array was validated. Finally, the array was used for gesture recognition of a limited set of gestures.

### 2.1. Sensor Development

The development of our sensor happened in two stages. In the first stage, a single, integrated, wireless sEMG sensor was developed, and in the second stage, this sensor was used to develop a sensor array. The different stages are outlined in the following subsections.

#### 2.1.1. sEMG Signals

Prior to the development of our sensor, we used a commercial reference sensor, the Myoware 1.0 (Advancer Technologies, LLC, Raleigh, NC, USA). In order to quantify what raw sEMG signals could be expected, we executed a set of reference measurements, where the Myoware was placed in different locations on the upper arm while doing several of the reference gestures from the Non-Invasive Adaptive Prosthetics (NinaPro) reference database [25]. From these preliminary measurements, we concluded that unamplified, an electromyographic biopotential has a range of −5 mV to +5 mV, centered around a reference electrode. The signal has a frequency range of 500 Hz, with the highest concentrated power between 50 Hz and 150 Hz. Following these specs, we then developed our sensors, whose performance was a posteriori validated. 

#### 2.1.2. A Wireless, Integrated sEMG Sensor

**Figure 1:**
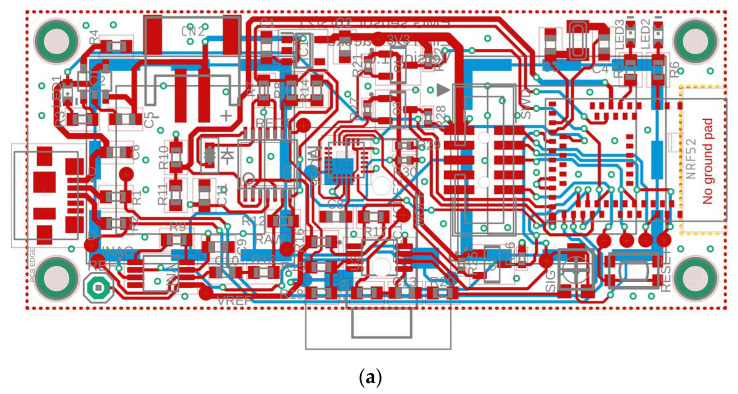

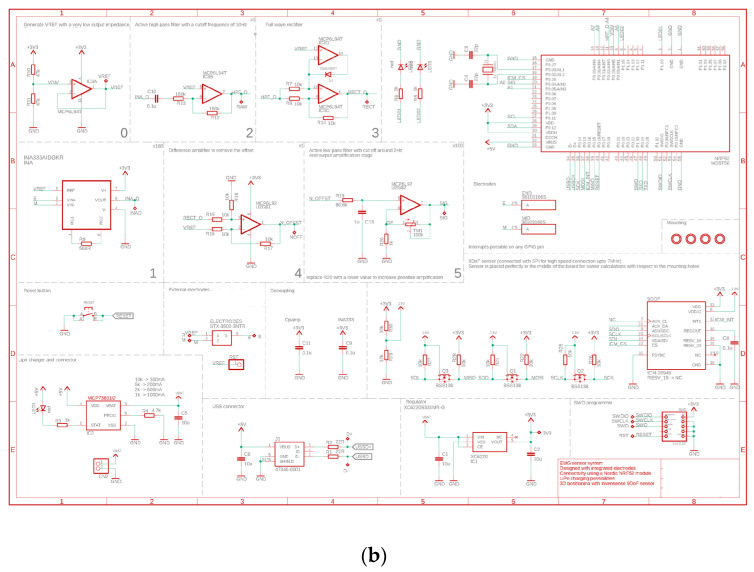
(**a**) Layout and (**b**) Schematic of the proposed wireless, integrated sEMG Sensor.

The goal of this prototype was to demonstrate a wireless system that could record sEMG signals. To this aim, we have designed an sEMG system fully integrated using a single Printed Circuit Board (PCB), which includes built-in electrodes and a Bluetooth SoC, making it a completely wireless system that can be connected to a battery. Figure 1 shows the proposed layout and schematic of the sensors. Each of its components is discussed below.

The sensor is powered by a rechargeable lithium-ion polymer battery (Lipo) with a capacity of 2000 mAh and a nominal voltage of 3.7 V. This battery has a relatively large energy density of 100–200 Wh/kg [26]. It is paired with a linear voltage regulator, a so-called low-dropout regulator (LDO), that can regulate the DC voltage to a value very close to the required input voltage of the system. The LDO used in this system is the XC6220 (Torex Semiconductor, Tokyo, Japan), which has a 3.3 V regulated voltage and a drop-out voltage of 50–350 mV. This LDO can handle a maximal load of 1 A, which is sufficient for this application. The sensor works with an asymmetric power supply from 0 to 3.3 V. Therefore, an offset voltage of 1.65 V was added to the sEMG signal voltage measurements to center them within the range of the supply voltage. This enabled us to reach the targeted sEMG voltage amplitudes between −5 mV and 5 mV.

**Figure 2:**
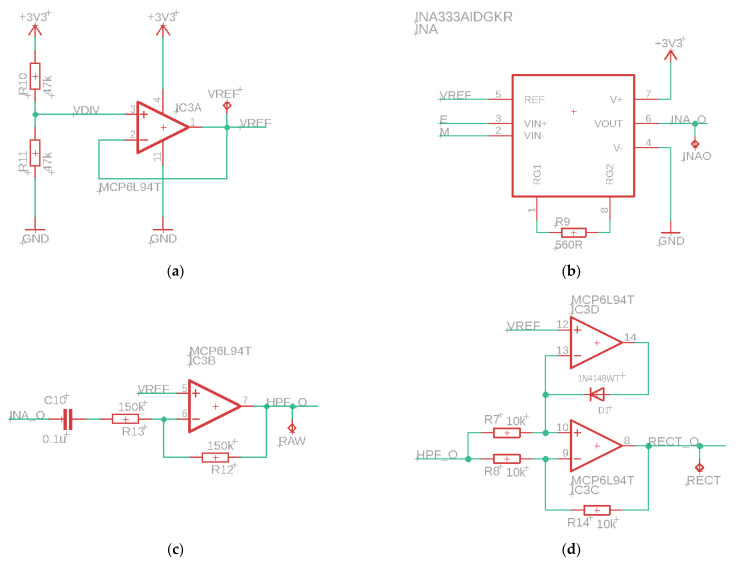

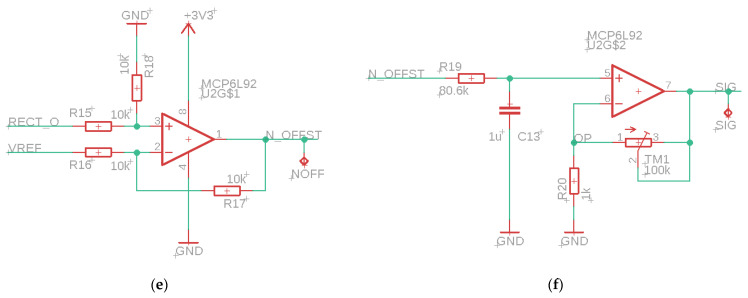
Electrical scheme of the parts of the sEMG sensor. (**a**) Reference voltage for offset, (**b**) instrumentation amplifier, (**c**) active high-pass filter, (**d**) bridge rectifier, (**e**) differential amplifier, (**f**) active low-pass filter with tunable amplifier.

The sEMG sensor consists of several parts, see Figure 2. First, an instrumentation amplifier (INA, INA333, Texas Instruments, Dallas, TX, USA) as input. This is a differential amplifier with a high Common Mode Rejection Ratio (CMRR, 100 dB) and adjustable gain, which is set after calibration of the sensor. The high CMRR of the INA also removes any 50 Hz noise. The INA is followed by an active high-pass filter with a cut-off frequency of 20 Hz, which filters out low-frequency noise due to motion artifacts. This is followed by a rectifier and a differential amplifier, which removes the offset voltage by subtracting the reference voltage from the measured signal. The last part of the circuit is an active lowpass filter with adjustable gain. The cutoff frequency of this filter is equal to 2 Hz. This filter is used to obtain the envelope of the sEMG signal.

The sensor also uses a Nordic nRF52840 Bluetooth SoC, which is an IC that contains a 32-bit ARM Cortex-M4 processor, 1 MB flash storage, and 256 kB RAM. The SoC also has Timers and a built-in Universal Serial Bus (USB) interface, which can be used to configure the IC and serial communication modules [27].

In terms of electrodes, we chose to work with dry electrodes rather than gel electrodes because they are reusable and require less skin preparation. They can also be mounted to the sensor permanently. These were mounted on the bottom of the sensor, where they can be in contact with the skin. To realize these electrodes, we used a conductive component that is normally used as a shielding component (36103166S by Wurth Elektronic, Künzelsau, Germany), shown in Figure 7b. To obtain a homogeneous and larger contact area between the electrode and the skin, we applied a layer of copper tape to cover the holes. The dry electrodes measured 16.1 × 16.1 × 3.6 mm^3^. Prior research conducted in the Surface ElectroMyoGraphy for the Non-Invasive Assessment of Muscles (SENIAM) project [28] recommends a size of 10 × 10 mm^2^ for dry electrodes. However, our tests showed that our larger electrodes did not present an excessive resistance for the sensor to work. Additionally, we also included a potential connection for gel electrodes in case one would prefer to work with such electrodes. In this case, the dry electrodes would need to be desoldered.

Because our sensor is used in direct contact with the skin, it was necessary to design a casing to shield the electronics from the skin. The design (30 × 64 × 12 mm^3^) of this case is shown in Figure 7c. The case is designed in such a way that the electrodes can be in direct contact with the skin while all other components remain shielded inside the case. 

#### 2.1.3. A Wireless, Integrated sEMG-Sensor Array

**Figure 3:**
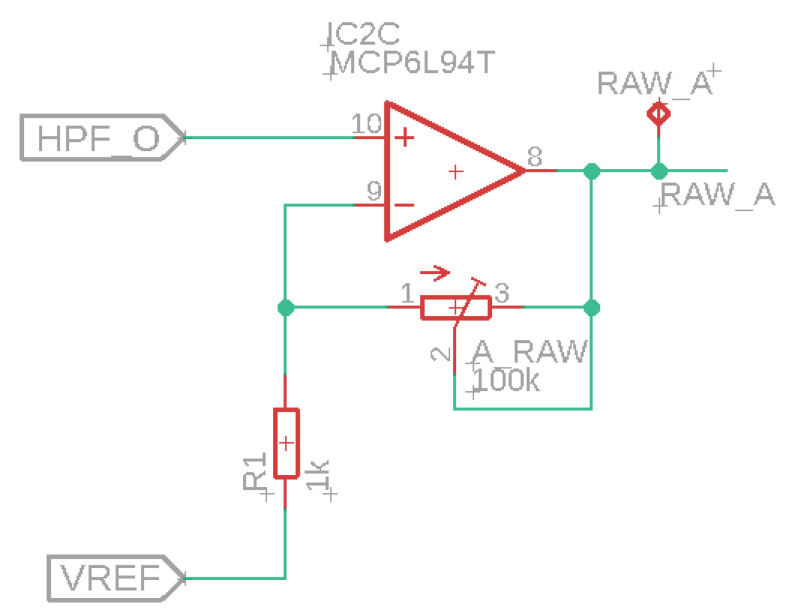
Amplification for raw sEMG signals.

In the next step, we developed a sensor array, which builds upon the wireless sensor described in the previous subsection. The main difference with the previous system is the addition of a bus system. The purpose of this system is to connect multiple sEMG sensors. It is not necessary for every node in the system to have a microcontroller, battery, and voltage regulator; i.e., it would be overly complex to create an array with a series of copies of the wireless sensor presented in the previous subsection. Therefore, we chose to work with an array with two types of nodes: a primary node, based on the prior design including the nRF52840 SoC, and a set of secondary nodes, which have fewer components, i.e., only the sEMG sensor and supporting electronics and whose sole function is to pass on the sEMG signals to the primary node. The sEMG sensor of the secondary node is the same as the one used in the primary node (and the sensor discussed in the previous paragraph). However, the secondary node uses a different circuit for the adjustable amplifier for the raw sEMG signals, which is shown in Figure 3.

**Figure 4:**
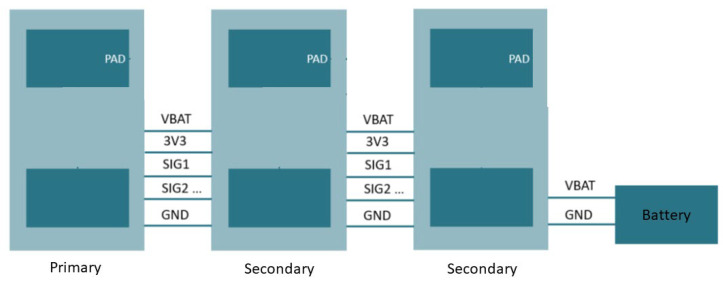
Parallel bus system for sEMG array.

The connections between the different nodes are outlined in the scheme shown in Figure 4. It depicts an example configuration of the array with a primary node connected to two secondary nodes. The entire system is fed by a single battery. The bus can support a maximum number of 5 secondary nodes, which was a choice that was made between the potential number of useful sensors and the size of the bus. The nRF52840 SoC could support eight channels in its analog to digital channel. This implies that the system could be extended to eight sensors if necessary (and potentially more if the sensors measure in series instead of parallel). The bus distributes the feeding voltage, the battery voltage, and the ground (the reference) to all nodes and also brings the sEMG signals from the secondary nodes toward the primary node. We chose to work with a parallel bus configuration because this allows for simultaneous measurement of sEMG in different locations.

### 2.2. sEMG Measurements

All test measurements with the sensors developed in this manuscript are executed with gestures as described in the Non-Invasive Adaptive Prosthetics (NinaPro) reference database [25]. This database [25] was developed with the aim to support research into upper arm prostheses.

**Figure 5:**
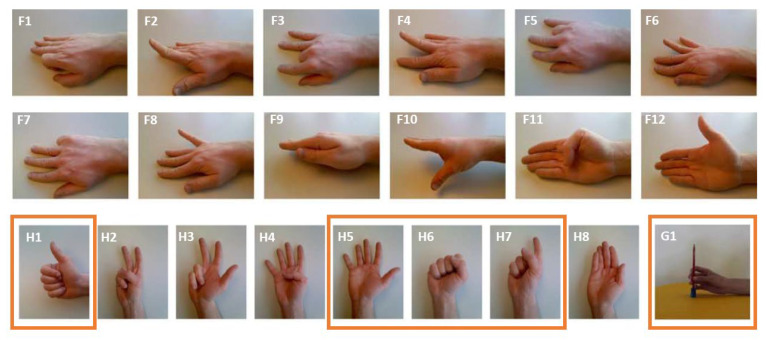
The Twenty-one gestures used in this research, selected from the NinaPro database [25]. The gestures indicated in an orange box are the ones used for gesture recognition.

Figure 5 lists the twenty gestures that are used throughout this manuscript. We limited ourselves to twenty gestures because this work is focused on sensor design rather than gesture recognition. We have labeled gestures only involving finger movements with an ‘F’ and only involving hand movements starting with the letter ‘H’, followed by an index *i* (e.g., F1, F2, …, F12 and H1, H2, …, H8), see Figure 5 [25]. We have also considered one grasp movement and denoted it G1. During all our measurements, a test subject formed all or a subset of the selected NinaPro gestures while the sEMG signals were recorded during, prior to, and after movement by the sensor(s). The location of the sensors on the arm, the protocol used for the measurements, and the number of gestures measured were different, depending on the goal of the measurements. Specific details on each type of measurement are provided in the following sections.

Every sEMG measurement results in a series of voltages measured by the sensor(s) over time $v_{jk}$ with *j* an index indicating the number of the sensor in the array and *k* an index indicating the temporal steps. After each measurement, we manually cleaned our data and split each measurement of a gesture into “rest” and “gesture” data. From this data, several features can be extracted from the time series of this voltage. We have chosen to work only in the time domain in this study since this reduces the computational requirements in the system; as Table 1 shows, other authors have used frequency-domain information from sEMG measurements as well. The features extracted from the data from sensor *j* are mean absolute value ($V_{MAV,j}$), root mean square ($V_{RMS,j}$), its variance (VAR($v_{j}$)), and the amount of slope sign changes ($N_{SSC,j}$) were calculated on a gesture per gesture basis, using:

(1) $$V_{MAV,j}=\frac{1}{K}\sum_{k=1}^{K}|v_{jk}|$$

(2) $$V_{RMS,j}=\sqrt{\frac{1}{K}\sum_{k=1}^{K}v_{jk}^{2}}$$

(3) $$\mathrm{VAR}(v_{j})=\frac{1}{K}\sum_{k=1}^{K}{\left({{\bar{v}}_{j}-v}_{jk}\right)}^{2}$$

With ${\bar{v}}_{j}$ the mean voltage recorded during the gesture by sensor *j* and K the number of samples (=100 in this manuscript). Prior to calculating $N_{SSC,j}$, a temporal averaging of 100 ms was applied to $v_{jk}$ giving ${\tilde{v}}_{jk}$. A slope sign was counted when the sign of ${\tilde{v}}_{jk}-{\tilde{v}}_{jk-1}$ is the opposite of the sign of ${\tilde{v}}_{jk+1}-{\tilde{v}}_{jk}$ for $k\in \left[2,\;K-1\right].$

### 2.3. Sensor Benchmarking

The commercial reference sensor used to compare our sensor was the Myoware 1.0 (Advancer Technologies, LLC, Raleigh, NC, USA), which measured the raw sEMG signal with a sampling frequency of 1 kHz. 

The measurement protocol for sensor benchmarking was the following. Twenty finger movements and hand gestures (F1 to F12 and H1 to H8, see Figure 5) were measured with our sensor and the Myoware sensor, placed on either the musculi flexor digitorum superficialis or the musculi extensor digitorum. The former is located on the abdominal side of the arm and is used for the flexion of all fingers except the thumb. The latter is located on the dorsal side of the arm and is used for the extension of all fingers except the thumb. These muscles were chosen because they are surface-level muscles and are required for most of the gestures [25]. During these measurements, the same subject executed the same set of gestures in the same order with either our sensor or the Myoware sensor placed in the same location on the arm. Each gesture always departs from a resting position, is held for a second, and then released. This is repeated fifteen times for each gesture before another gesture is started. This protocol is inspired by the protocol of [25]. To benchmark our sensor, the root-mean-squared voltage ($V_{j,RMS})$ was calculated on a gesture per gesture basis, using Equation (1) This results in a group of 15 values for each gesture and for each sensor j. The median is then calculated for each gesture, and the Spearman correlation (r^2^) is calculated for the two sets of medians. A high correlation between median $V_{j,RMS}$ indicates that the sensors can be interchanged to measure the same quantity during these gestures. 

### 2.4. sEMG Array Validation

To demonstrate the usefulness of our sensor array, we executed sEMG measurements with the array during 16 gestures (F1–8 & H1–8) shown in Figure 5, using the same protocol and placement as described in Section 2.3. $V_{RMS,j}$ were again calculated for each sensor node *j* within the array. The median $V_{RMS,j}$ values for each gesture as measured by two sensors, one on the abdominal side of the arm and one on the dorsal side were also used to calculate a Spearman correlation coefficient. Here, an r^2^ close to 0 indicates that both sensor nodes are uncorrelated during the same gesture, which means that they provide uncorrelated information to a potential gesture recognition algorithm, which improves its accuracy [7].

### 2.5. Gesture Recognition

Our final goal was to demonstrate that the sEMG-sensor array developed in this manuscript could be used for gesture recognition. To this aim, a set of measurements was carried out, which was used to train a machine learning protocol, which was then used to classify test data. The following subsections outline the method used in this demonstration.

#### 2.5.1. Sensor Configuration, Placement, and Data Acquisition

**Figure 6:**
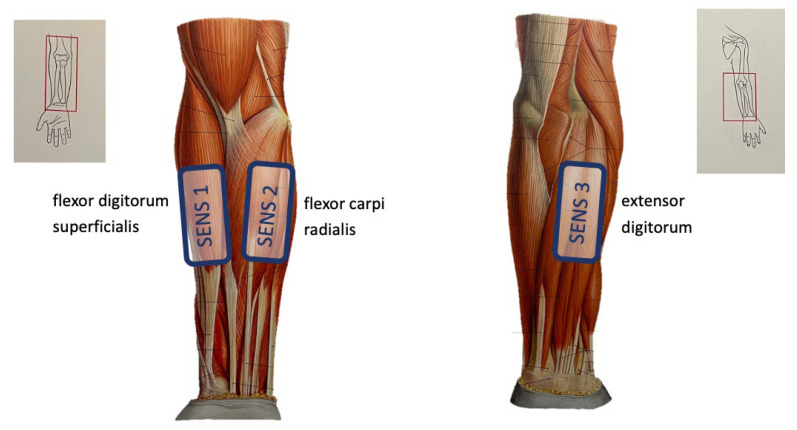
Illustration of sensor placement during the gesture recognition demonstration.

The data acquisition protocol for gesture recognition is based on the NinaPro protocol [25]. First, the subject sits comfortably with their dominant arm resting on a table. Second, the sEMG sensors are placed on the specified muscles, see Figure 6. To ensure good contact between the sensors and the skin, the elastic band that keeps the sensors around the forearm is tightened. Third, good sensor placement is verified by comparing trial sEMG measurements with expected sEMG values. Different gestures are performed, and it is checked whether the sEMG signals are stable and the measured potentials follow the movement performed. Fourth, the subject goes into a resting state, from which each gesture shall start. Each gesture shall be performed 30 times for 5 s, with a 3 s resting period in between the repetitions in order to prevent muscle fatigue. During the first phase of a gesture (∼1 s), there is a transition from rest into the desired gesture. This position is then held during 3 s, and then there is a relaxation period into rest of ∼1 s. Finally, before transitioning into the next set of repetitions of another gesture, there is a resting period of 1 min. This sequence is accompanied by a video that shows which gesture needs to be performed and the speed of the movements, as well as the rest periods, as described above. The gesture classification was only executed for a subset of the gestures shown in Figure 5, namely {rest, H1, H5, H6, H7, G1}. Hence, the protocol resulted in 30 repetitions of 6 gestures for 5 s.

During these measurements, the sensor array was configured to work with 3 nodes and recorded samples with a frequency of 1 kHz. One was placed on the flexor digitorum superficialis, which controls the flexion of the index fingers, and a second sensor was placed on the musculi extensor digitorum, which controls the extension of the index fingers. The third sensor was placed in between these two main muscles on the flexor carpi radialis, which controls wrist flexion and wrist abduction. Figure 6 illustrates the placement of the 3 sensors.

#### 2.5.2. Data Processing and Machine Learning for Gesture Classification

Measurement data obtained using the protocol described in the previous section was stored on a laptop on a gesture-per-gesture basis and post-processed in Python. The data was labeled by a researcher to categorize the data in the right gesture categories prior to the training. All features listed in Section 2.2 are calculated for all repetitions of all gestures for a time window of 100 ms, which gives 50 data points per feature per repetition of a gesture of 5 s. In order to clean this data for demonstration purposes, the first and final 15 samples for each feature were discarded. This choice was the result of an optimization, where the classifier was trained using different lengths of censored data at the start and stopping of a movement. After that, all features were scaled by subtracting the mean and scaling the feature set to unit variance. We want to point out that this post-processing is in no way relevant to a real-life application of gesture recognition. However, it is solely meant to demonstrate that our sensor could be used for such purposes.

We initially trained a ridge classifier, a decision tree algorithm, a logistic regression model, and a support vector machine alongside a random forest classifier to check initial performance prior to secondary hyperparameter tuning. The random forest classifier performed best in this case and was then further trained on the features of the six gestures. A random forest is a type of ensemble learning algorithm that combines multiple decision trees. More information on this type of classifier can be found in [29]. 

During the training, a stratified group split with a ratio of 80 to 20 for training and testing was used. An equal distribution of all gestures is used for gesture recognition in both the training, i.e., 24 movements of each gesture in the training set and 6 movements of each gesture in the testing set. While training the classifier, a 5-fold cross-validation is performed to tune the model’s hyperparameters. The total amount of movements (24 × 6 gestures) is split into 4 folds of 29 movements and 1 fold of 28 movements in a stratified manner with 4 or 5 movements from each gesture in each group.

## 3. Results

This section presents the different sensor prototypes, the validation and benchmarking of the sensors, and the application of the sensor in gesture recognition.

### 3.1. sEMG Sensors

The development of our sensor happened in two stages. In the first stage, a single, integrated, wireless sEMG sensor was developed, and in the second stage, this sensor was used to develop a wireless sensor array. The resulting sensors are shown in the following subsections.

#### 3.1.1. A Wireless, Integrated sEMG Sensor

**Figure 7:**
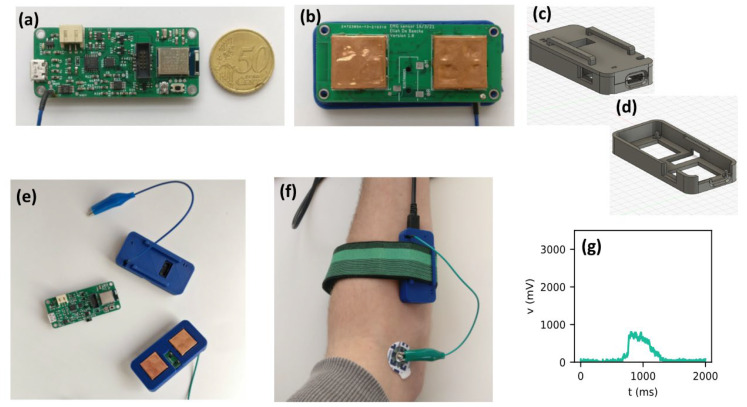
(**a**) Top view of wireless sEMG sensor with 50 euro cent piece as a reference (58 × 25 × 12 mm^3^), (**b**) Bottom view of the same sensor with electrodes and potential connection for gel electrodes; sEMG sensor case with (**c**) and without (**d**) cover (**e**) Sensor with electrodes in casing, (**f**) Sensor during sEMG measurements, and (**g**) An example of a measurement of a rectified sEMG voltage during gesture F5.

The resulting system is shown in Figure 7, where (a) to (d) show the sensor and how it can be used during wireless sEMG measurements. Figure 7e shows a wirelessly transmitted sEMG measurement during gesture F5, demonstrating that the sensor can read sEMG signals, even for relatively small finger movements.

In order to test the wireless performance, the Bluetooth SoC was programmed to wirelessly transmit the measured sEMG signal to a connected device when a muscle contraction is detected. A maximum transmission rate of 125 kB/s was achieved. Since sampling is conducted at 1 kH, and 12 bits are needed per value (max voltage of 3300 mV), the transmission rate of 125 kB/s should suffice to stream measurements to a prosthetic arm, which can then be processed.

#### 3.1.2. A Wireless, Integrated sEMG-Sensor Array

**Figure 8:**
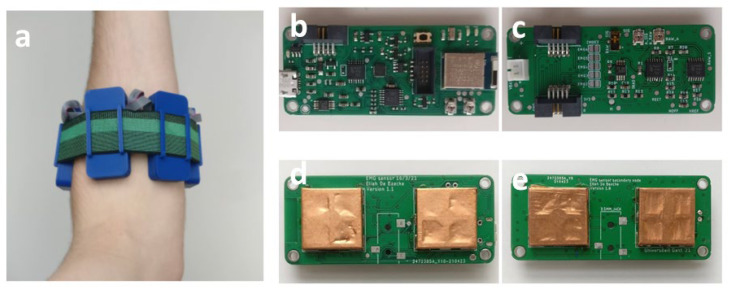
A primary and three secondary nodes form the sEMG array. (**a**) Placed on the left forearm, top view of (**b**) primary node and (**c**) secondary node, bottom view of (**d**) primary node and (**e**) secondary node (node size: 58 × 25 × 12 mm^3^).

Figure 8 shows a realization of the array with one primary node and three secondary nodes placed on the left forearm of a subject. The PCBs that form the sEMG array were assembled manually using a reflow oven and SMT stencils. The finished PCBs are shown in Figure 8b–e. The array is designed to either work with an external reference electrode, such as shown in Figure 7d, or to rotate the reference electrode from one electrode to the next during subsequent measurements. However, in the following measurements, this was not necessary because we maximally used three electrodes. Hence, one of them was always used as a reference.

### 3.2. Sensor Benchmarking

**Figure 9:**
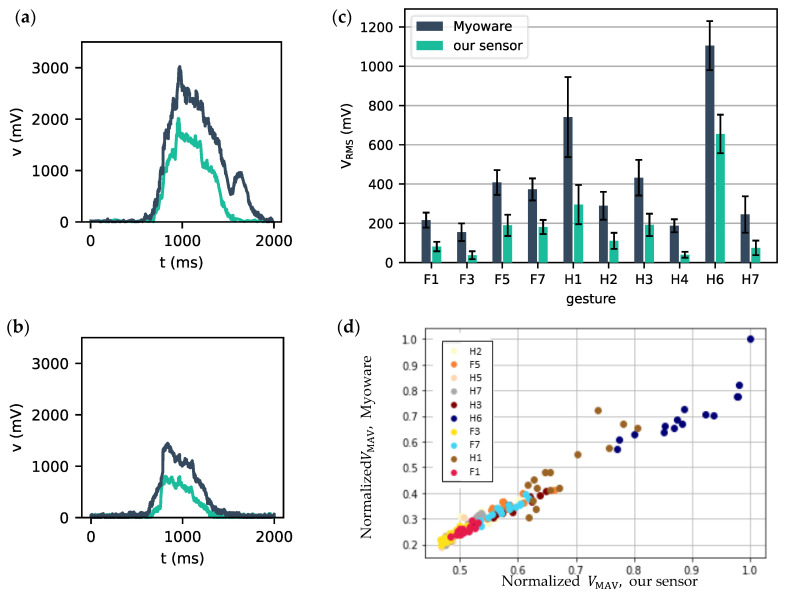
Measured sEMG potentials for gestures (**a**) H6 and (**b**) F5 with our sensor (green) and the Myoware sensor (blue). (**c**) Mean root-mean-squared voltage was measured with our sensor (green) and the Myoware sensor (blue) for 10/20 studied gestures. Standard deviations are depicted with error bars. (**d**) Normalized V_MAV_ for the Myoware and our sensor for 10 gestures, markers with the same colors indicate repetitions of the same gesture, while a different color indicates a different gesture.

In this section, our single, wireless, integrated sEMG sensor is compared to the commercial Myoware sensor. Figure 9 shows that there is a clear temporal relationship between the measurements of the two sensors. The measured potentials with both sensors show very similar temporal profiles for the same gestures. The amplitudes obtained for the Myoware are probably higher because it uses more sensitive wet electrodes. However, we could match this by tuning our amplifier. The signals shown here are higher than what is normally expected for raw sEMG; this is also due to the amplification in our sensor. When comparing how V_RMS_ varies from one gesture to another in Figure 9c, a very similar trend in mean gestures can be observed. The median V_RMS_ over all repetitions of each gesture was calculated for each sensor, leading to two sets of 20 paired values, for which a Spearman correlation of 0.98 (*p* = 1.5 × 10^−6^ < 0.01) was found. Indicating that the results from the two sensors are highly correlated.

However, Figure 9c also depicts a clear difference in absolute signal voltage amplitude. This is mainly explained by a higher resting voltage of the muscles for our sensor, i.e., 370 mV, compared to 170 mV for the MyoWare. A higher resting voltage leads to a lower dynamic range of the sensor, which in turn leads to lower amplitudes being recorded when the resting voltage is subtracted. A mitigation strategy for this would be to calibrate the resting voltage prior to starting the measurements. 

To further illustrate the excellent correlation between both sensors, Figure 9d shows pairwise plots of normalized V_MAV_-values of our sensor versus the MyoWare sensor. These values were normalized to the maximally recorded V_MAV_ pair (1,1) on the top right corner. This normalization was used for muscle and gesture-specific amplitude offsets. Uncorrelated measurements would result in a scatter cloud between 0 and 1, while perfectly correlated measurements would all lie on the bisector. It is clear from this figure that both sensors are highly correlated and that measurements coming from the same gesture are grouped.

### 3.3. sEMG Array Validation

**Figure 10:**
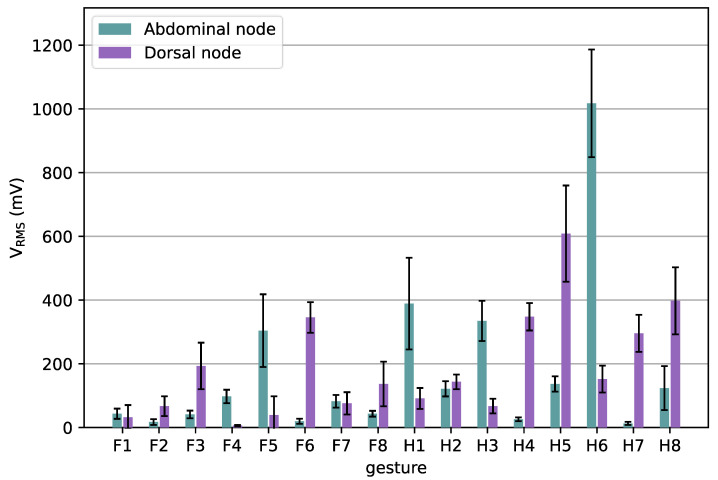
Mean_VRMS_ of two nodes the sEMG array as a function of the gestures. The whiskers indicate standard deviations.

To validate the performance of the sEMG array, 16 gestures were measured with the array. The measurements from the node on the abdominal side of the arm and the one on the dorsal side were further processed and compared. Figure 10 shows the mean V_RMS_ measurements as a function of each gesture. In Figure 10, we have limited the number of sensors to two to clearly show the difference between sEMG signals from dorsal and abdominal muscles. The movements that only require the index fingers, F1 & F2, show only a small V_RMS_, which was also the case for the MyoWare and our wireless prototype, see Figure 9. Other gestures show a relatively big difference between the sensor on the dorsal and the abdominal side of the arm. Gestures showing greater flexion, e.g., F5, H1, and H6, show higher V_RMS_ on the abdominal side, while gestures with greater extension, such as F6, H4, H5, and H8, have greater signal amplitudes at the dorsal sensor, as was expected. 

The Spearman correlation between the median V_RMS_ values measured for the twenty measured gestures (all shown in Figure 5 except G1) resulted in a correlation of R = −0.14 (*p* > 0.62), which indicates that the measurements between the abdominal and dorsal nodes are nearly uncorrelated. The use of uncorrelated data can improve the performance of classifiers for gesture recognition [7].

### 3.4. Gesture Recognition

Initial exploration of different classifiers on the same set of 6 gestures, as described in Section 2.5, led to validation accuracies of 89 ± 1.9% (ridge classifier), 88 ± 2.4% (decision tree), 89 ± 1.7% (logistic regression), 91 ± 1.9% (support vector machine), 92 ± 2.1% (random forest).

**Figure 11:**
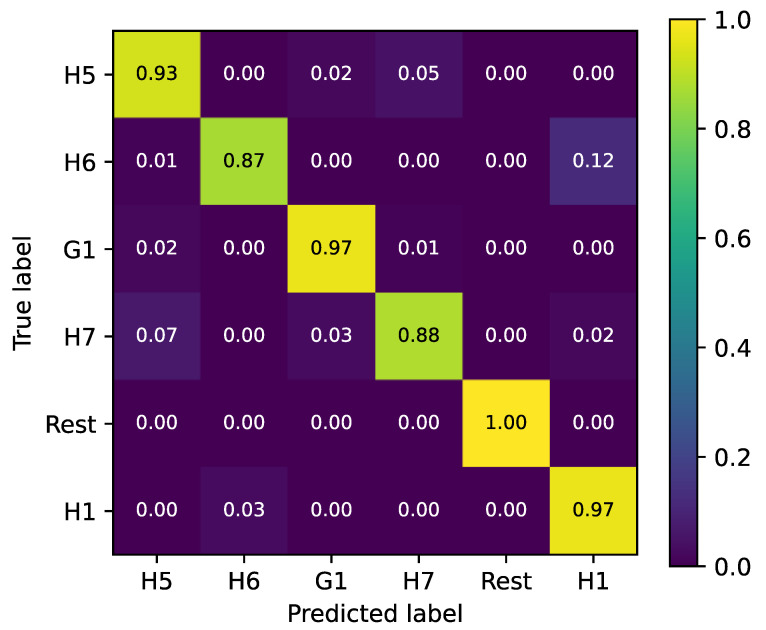
Confusion matrix of random forest classifier after hyperparameter tuning. The color bar indicates the accuracy of the classifier with 1 indicating 100% accuracy and 0 indicating 0% accuracy.

Therefore, we further trained a random forest classifier on a set of 6 gestures, as described in Section 2.5. The training of the random forest classifier involves tuning the classifier’s hyperparameters. First, the amount of trees in the forest was tuned, for which an optimum of 132 trees was found. Second, the maximum depth of the algorithm was tuned, for which an optimum of 20 layers was found. The final hyperparameter that was tuned is the minimum amount of samples a node needs to make a split on. After setting the third parameter at 10, a final hyperparameter tuning with a small grid search was performed for the first two hyperparameters, and no improvements were found for alternate values. The final test accuracy was 96.8 ± 2.2%. Figure 11 shows the confusion matrix of the final classifier. The main confusions are found between the H7- H5 and H1-H6. 7% of pointing movements (H7) are classified as ‘five’ (H5), and 5% of ‘five’ as ‘pointing’. There are also 12% of first (H6) movements being misclassified as thumbs up (H1), while 3% are misclassified the other way around. 

### 3.5. Cost

There are four main factors that determine the price of an sEMG sensor: (i) PCB manufacturing costs, which in this case were less than €5; (ii) cost of components, which was €45 and €15 for the primary and secondary nodes, respectively, (iii) cost for PCB assembly are less than €30, (iv) costs for printing the case for the sensor, which is less than €1 per case. All costs were determined in June 2021.

## 4. Discussion

The results presented in the prior section demonstrate that our sEMG array is functional and can be used for gesture recognition. However, the array could still be improved. A drawback of a circular sEMG array mounted around the arm is that the muscles that are located in the hand itself, such as those controlling thumb movement, cannot be measured directly. Our sensor should be able to measure indirect signals from wrist and hand movements associated with a thumb movement, for example, with its inertial sensor. However, as Figure 11 shows, it is difficult for the array to distinguish between the “thumbs up” and “fist” gestures, which are only distinguishable by the position of the thumb. A thumb-specific sensor could aid in mitigating this for gesture recognition of non-amputees, but for any upper limb amputee, this will remain a problem. Due to the small footprint of our sEMG nodes, the sensor can be worn on different arm locations without any issues in terms of comfort. However, for placement around the wrist (wrist-band sEMG), a smaller footprint would be necessary. This could then also be used to decrease the size of the electrodes. Another potential improvement would be to use flexible PCBs for a more ergonomic design that can follow the arm’s curvature. A final design improvement would be to make the case of the sEMG nodes waterproof. Technically, our sEMG sensor could be improved by working with a feedback loop that can adapt the DC offset in our system to correct positional variations. At this moment, we are working with a parallel bus system to connect the electrodes within the array, which limits the total number of sEMG sensors within the bus. An alternative would be to work with a serial protocol such as the Inter-Integrated Circuits- (I2C) of the Controller Area Network (CAN) protocols. We have mainly analyzed our sensor in the time domain because most papers use TD features for classification, see Table 1. However, some papers use frequency domain (FD) features as well (Table 1). Therefore, a good next step in this research would be to also analyze the FD performance of the sensor. A disadvantage of working with our system, in comparison to wired and/or analog sensors, is that we use analog-to-digital conversion to transmit the sEMG data digitally and wirelessly. This might lead to packet loss or bit errors, which could introduce errors in the frequency domain that would not be there in a wired or fully analog solution. 

For gesture recognition, we found a final accuracy of 96.8 ± 2.2%, which is comparable to what is found in literature in cases with a comparable number of sensors, quality of sensors, sample rate, and number of gestures. Junior et al. [20] found an accuracy of 94% for five gestures with eight sensor channels measured at 2 kHz. Leone et al. [21] obtained an accuracy of 99% in classifying six gestures using six sensor channels measured at 1 kHz. Finally, Oskoei et al. [24] classified five hand gestures using four sensors measuring at 1 kHz with an accuracy of 96%. Further improvements in classification could be made by exploring other classifiers and using different features, including classification post-processing, a higher sampling rate, sensor placement optimization, and using an array with more channels. An alternative protocol for obtaining training data could be considered since the current protocol involves transitions between gestures that could lead to misclassification if the data is not cleaned beforehand. An alternative to this approach would be to use the transitions between the different gestures as separate gestures themselves and include them in the classifier. The next step in this research would be to repeat this process on multiple subjects to study inter-subject variability and train the classifier to either deal with this or to work with subject-specific training.

We have shown that our sensor can be used to measure sEMG signals and that these can be wirelessly submitted to a computer on which gesture classification can be conducted. Wireless, Bluetooth-controlled prosthetic arms have been demonstrated in the literature [30]. Hence, it would be possible to control such an arm using a wireless connection from our sensor to a computer and a simultaneous wireless connection from a computer to such a prosthesis. We are currently developing a wireless system for integration with an open-source prosthesis, which could directly connect to our sensor, perform gesture classification, and transmit this to the prosthesis’s existing control electronics.

## 5. Conclusions

Affordable arm prostheses and systems to control them are needed by many patients worldwide. Surface Electromyography (sEMG) using capacitive electrodes is a good method to enable intuitive control of arm prostheses. The wiring that is involved in connecting such sEMG systems to a prosthesis is unwanted by patients. Single sEMG nodes do not provide enough information for gesture classification. Therefore, we developed two systems: a wireless sEMG node and a circular sEMG array. The sensors rely on Bluetooth to wirelessly transmit measurements. We showed that this prototype measures signals during a set of standardized gestures that are highly correlated with those that can be measured using a wired commercial alternative. We also showed that the difference in measured biopotentials is in line with expectations based on anatomical knowledge of the muscles that are involved in the measured gestures. We show that an array with our sEMG nodes can yield uncorrelated yet accurate data for a set of 20 gestures. A random forest classifier is built to classify six gestures measured by the circular EMG array with three sensors. These gestures are measured according to the Ninapro protocol. An accuracy of 97% was obtained, which is on par with the state of the art for wired, low-cost sEMG sensors.

## Figures and Tables

**Table 1:** Overview of previous research on gesture classification for sEMG with multiple channels.

# Sensor Channels	Sampling Rate [kHz]	Gestures	Classification Method	Used Features	Accuracy	Reference
8 pairs (bipolar)	1.2	8 wrist gestures	Convolutional neural network (CNN, 22 layers)	Raw sEMG data	91.61 ± 0.39%	[18]
8 pairs (bipolar)	1.2	8 wrist gestures	Support vector machine (SVM)	Time-Domain (TD) Features and mean frequency	90.63 ± 0.31%	
10	1	10 hand/wrist gestures	Deep neural network (DNN)	Learned features	84.43 ± 0.05%	[19]
8	2	5 wrist gestures	Large margin nearest neighbours	TD features and sample entropy	94%	[20]
6	1	7 hand/wrist gestures	Logistic regression	Raw sEMG data	98.78%	[21]
8	0.2	grasp gesture (8 force levels)	DNN	4 TD features	>95%	[22]
16 × 4	1	21 hand/wrist gestures	Hyperdimensional computing	Mean absolute value	84.53%	[7]
16 × 8	2.048	16 hand/wrist gestures	CNN with 7 layers	Learned features	78.7 ± 0.08%.	[23]
4	1	5 hand gestures	SVM	TD features and Frequency Domain (FD) features	95.5 ± 3.8%	[24]
12	2	41 hand and wrist gestures (Ninapro)	CNN (3 layers)	TD features	84.66 ± 4.78%	[6]

## Data Availability

Data are contained within the article.
